# Using Object Oriented Bayesian Networks to Model Linkage, Linkage Disequilibrium and Mutations between STR Markers

**DOI:** 10.1371/journal.pone.0043873

**Published:** 2012-09-11

**Authors:** Daniel Kling, Thore Egeland, Petter Mostad

**Affiliations:** 1 Department of Family Genetics, Norwegian Institute of Public Health, Oslo, Norway; 2 Department for Chemistry, Biotechnology and Food Science, Norwegian University of Life Sciences, Aas, Norway; 3 Mathematical Sciences, Chalmers University of Technology and Mathematical Sciences, Gothenburg, Sweden; Queen's University Belfast, United Kingdom

## Abstract

In a number of applications there is a need to determine the most likely pedigree for a group of persons based on genetic markers. Adequate models are needed to reach this goal. The markers used to perform the statistical calculations can be linked and there may also be linkage disequilibrium (LD) in the population. The purpose of this paper is to present a graphical Bayesian Network framework to deal with such data. Potential LD is normally ignored and it is important to verify that the resulting calculations are not biased. Even if linkage does not influence results for regular paternity cases, it may have substantial impact on likelihood ratios involving other, more extended pedigrees. Models for LD influence likelihoods for all pedigrees to some degree and an initial estimate of the impact of ignoring LD and/or linkage is desirable, going beyond mere rules of thumb based on marker distance. Furthermore, we show how one can readily include a mutation model in the Bayesian Network; extending other programs or formulas to include such models may require considerable amounts of work and will in many case not be practical. As an example, we consider the two STR markers vWa and D12S391. We estimate probabilities for population haplotypes to account for LD using a method based on data from trios, while an estimate for the degree of linkage is taken from the literature. The results show that accounting for haplotype frequencies is unnecessary in most cases for this specific pair of markers. When doing calculations on regular paternity cases, the markers can be considered statistically independent. In more complex cases of disputed relatedness, for instance cases involving siblings or so-called deficient cases, or when small differences in the LR matter, independence should not be assumed. (The networks are freely available at http://arken.umb.no/~dakl/BayesianNetworks.)

## Introduction

There are several areas of applications motivating this paper. The general problem is to determine the most likely pedigree and in this paper we discuss models to achieve this goal. It is well known that linkage analysis performed to locate disease mutations may be misguided if the pedigree is incorrectly specified as will be the case if for instance false paternities are not detected. Similarly, association analyses frequently assume that all individuals are unrelated and again deviations from this assumption may affect conclusions. In forensic cases, for instance paternity cases or identification following disasters, establishing the most likely pedigree is the main objective. Traditionally forensic applications have been based on unlinked markers in linkage equilibrium. For some applications however, these assumptions have been questioned [1], [2], [3] Furthermore, the conventional markers used in forensics may not have sufficient power to resolve some cases, e.g. family relationships involving more distant relations than siblings [4], [5], [6]. It is therefore an urgent need to consider methods and practical implementations for more general markers and this is the main objective for this paper.

The evidence is conventionally summarized by the LR (likelihood ratio) [7]. The LR is the probability of the data given one hypothesis (for instance that a specific man is the father) divided by the probability conditioned on an alternative hypotheses (for instance that some unknown man is the father). A large value of the LR results in a man being declared to be a father. In immigration cases, LR calculations can be decisive when decisions are made on granting immigration. It follows that biased LR calculations resulting from unwarranted assumptions may have serious consequences. As far as we know, methods and implementations accounting for linkage, linkage disequilibrium and mutation have not previously been presented.

A forensic example involving two short tandem repeat (STR) loci, D12S391 and vWa, will serve as a motivating case. These markers are located on chromosome 12 only 6.3 Mb apart, but the genetic distance has been estimated to be as large as 10.8 cM [3]. Following the introduction of D12S391 to the new European forensic standard set [8], questions has been raised as to whether the markers can be considered statistically independent when assessing the evidence in specific cases. In addition, studies have been performed to determine whether the physical proximity of the markers has caused linkage disequilibrium (LD) and whether this should be taken into account [1], [3]. Moreover, Phillips *et al.* recently published an overview of the commercial STR kits describing several pairs of markers separated by less than 50 cM [9]. Commonly used software for likelihood ratio calculations, such as Familias [10] and DNAView [11] do not consider linkage or linkage disequilibrium in statistical calculations. Although programs exist which model linkage, they are often more complicated to use and it may be necessary to navigate a command line user-interface, e.g. Merlin [12], [13]. In addition, to our knowledge, there is no complete model which simultaneously handles linkage, LD and mutations.

Object Oriented Bayesian Networks (OOBN) may provide an alternative solution with an appealing graphical interface. The object-oriented approach also provides a simple user-interface, hiding the complexities within the objects (nodes) [14]. In the model, the nodes contain sub-structures such as states, conditional probability tables and so forth. The nodes are connected to other nodes and the interplay is governed by probabilities within each node. Several studies have already shown the advantages of using OOBN in forensic contexts [15], [16], [17], [18], [19], [20]. Taroni *et al.* [15] offers a thorough introduction to the basic methodology. We used the freeware GeNIe (http://genie.sis.pitt.edu) to create the Bayesian networks. One alternative is the commercially available Hugin (http://www.hugin.com).

In this paper we model linkage, linkage disequilibrium, and mutations in a single Bayesian network (BN), freely available at http://arken.umb.no/~dakl/BayesianNetworks/. We present networks for some basic relationships, but the model can easily be extended to other pedigrees as well. In addition to previous investigations, this provides an alternative approach to the study of LD between D12S391 and vWa, but also more generally when studying pairs of linked STR markers. In contrast to other studies, which often measures the disequilibrium, or association of alleles, in terms of an *r^2^* value or a *p*-value depending on a sample size, our intention was to investigate the effects of LD on actual cases.

## Materials and Methods

In order to model linkage disequilibrium (LD), haplotype probabilities must be estimated. A simplified model was constructed (Tillmar *et al.* [21]), based on a Dirichlet distribution, providing non-zero probability estimates also for unseen haplotypes. Specifically, a diallelic haplotype probability 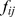 was estimated with 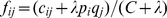 where 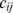 is the observed count of the haplotype among 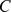 unrelated individuals, 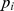 and 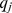 are the allele frequencies of the two alleles, and 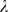 is a constant, set to 1 in the computations below. Further, to incorporate this into a Bayesian Network (BN) the haplotype probabilities were used to construct conditional allele probabilities, i.e. based on what allele is observed at the first locus we estimated the conditional probability of observing each allele at the second locus.

In order to obtain haplotype counts, we used data from regular trio paternity cases. When the parenthood is established and no mutations are present, the phase, i.e., the haplotypes can be deduced for the child using a simple algorithm. There are, however, ambiguous cases where the haplotypes cannot be determined for the child, e.g. when the parents and the child are all heterozygous for the same alleles. Out of 450 selected trios, 6 where discarded due to more than one possible haplotype configuration. As these ambiguous cases constitute only 1.3% of the total cases, it was not considered to bias the calculations enough to influence the conclusions. Notice that the phased haplotypes for the father and mother, based on the child's genotypes, are generally unknown since recombination might have occurred. Although reasonable estimates of the parents' haplotypes can be obtained, e.g. through the EM-algorithm or Gibbs sampling (PHASE by Stephens *et al.* and IMPUTE2 by Howie *et al.*, [22], [23]), we found that haplotype probabilities computed this way did not differ much from those based on the children and therefore used the latter for simplicity (data not shown). Moreover, it is well known that the LD as measured by *D* declines with (1-recombination rate) per generation and hence,one generation will only have a minor impact on the disequilibrium.

### Data

A selection of 444 unrelated Norwegian trios were used to estimate allele and haplotype probabilities at the STR loci D12S391 and vWa (using only the genotypes from the children). Table 1 describes the allele frequencies; in total 8 different alleles were observed at vWa and 16 different alleles at D12S391. To estimate haplotype probabilities, the number of observations for each haplotype was first counted (using the data from the children). In total, 100 different haplotypes were observed out of 128 possible. Haplotype probabilities were then estimated as described above. (Tables S1 and S2 provide further details on the observed haplotype frequencies and the estimated haplotype probabilities). To calculate the conditional probability of each D12S391 allele given a specific vWa allele, each column in Table S1, containing the observed haplotype probabilities, is normalized to 1. Table 2 describes the calculated conditional allele probabilities. Conditioning rather on D12S391 would of course lead to the same results.

**Table 1:** Sample allele frequencies for STR loci vWa and D12S391, based on 444 unrelated Norwegian individuals.

	vWa	D12S391
**14**	0.08896	
**15**	0.0732	0.04392
**16**	0.21621	0.02252
**17**	0.30968	0.12387
**17.3**		0.01351
**18**	0.1982	0.19369
**18.3**		0.01351
**19**	0.10248	0.10698
**19.3**		0.01126
**20**	0.10135	0.10811
**21**	0.00114	0.10248
**22**		0.01149
**23**		0.09234
**24**		0.03829
**25**		0.00901
**26**		0.00338
**27**		0.00225

**Table 2:** Conditional allele probabilities for the alleles at D12S391 given the allele at vWa.

	14	15	16	17	18	19	20	21
**15**	0.038049	0.030969	0.036497	0.043637	0.056745	0.054825	0.004392	0.021959
**16**	0.000282	0.030644	0.020842	0.029067	0.028376	0.011114	0.002252	0.011261
**17**	0.176548	0.062483	0.156082	0.112768	0.091095	0.131781	0.312387	0.061937
**17.3**	0.000169	0.000205	0.015614	0.018165	0.017026	0.011017	0.001351	0.006757
**18**	0.177421	0.199904	0.177169	0.207224	0.221433	0.154279	0.119369	0.096847
**18.3**	0.012669	0.015356	0.005251	0.014542	0.028325	0.000147	0.001351	0.006757
**19**	0.138837	0.062227	0.114544	0.09459	0.113599	0.131598	0.010698	0.053491
**19.3**	0.000141	0.015322	0.015602	0.018157	0.005713	0.000122	0.001126	0.005631
**20**	0.076351	0.168305	0.083462	0.090971	0.136204	0.142479	0.110811	0.054054
**21**	0.126281	0.153068	0.109339	0.098197	0.074025	0.09894	0.110248	0.051239
**22**	0.076437	0.122953	0.104222	0.14172	0.096694	0.109945	0.211487	0.057433
**23**	0.126154	0.062005	0.098924	0.083668	0.079618	0.109699	0.109234	0.546171
**24**	0.037979	0.061186	0.046831	0.032747	0.039764	0.022155	0.003829	0.019144
**25**	0.012613	0.015288	0.005228	0.010902	0.005701	0.010968	0.000901	0.004505
**26**	4.22E-05	5.12E-05	0.01038	1.22E-05	0.005669	3.67E-05	0.000338	0.001689
**27**	2.82E-05	3.41E-05	1.17E-05	0.003631	1.27E-05	0.010894	0.000225	0.001126

To account for unseen haplotypes, probabilities were estimated using a Dirichlet distribution. Each row indicates the allele at vWa, while each column indicates the allele at D12S391. The table should be interpreted as follows, for a given allele at vWa (top row), the corresponding conditional allele probabilities for D12S391 are given (column).

### Network

**Figure 1:**
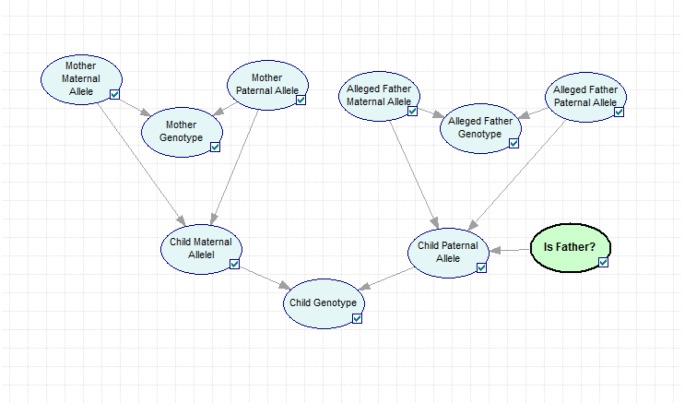
Bayesian network describing the basic layout for a paternity case.

A simple Bayesian network describing a paternity case is illustrated in Fig. 1, the network is more or less self-explanatory and presents the given problem in a intuitive way. It is worth pointing out that as more parameters (i.e. recombination rates, LD and mutations), markers and more distant relationships are considered, the network grows in complexity and can become visually incomprehensible. This can be counteracted by rearranging the most relevant network nodes in a simpler way, hiding the complexity from the user. The networks created in this study use a simple naming convention, based on few abbreviations, but larger networks might require shorter node names. All networks are freely available at http://arken.umb.no/~dakl/BayesianNetworks/. In addition we provide a short user manual as well as a software to generate the networks based on your own data.

**Figure 2:**
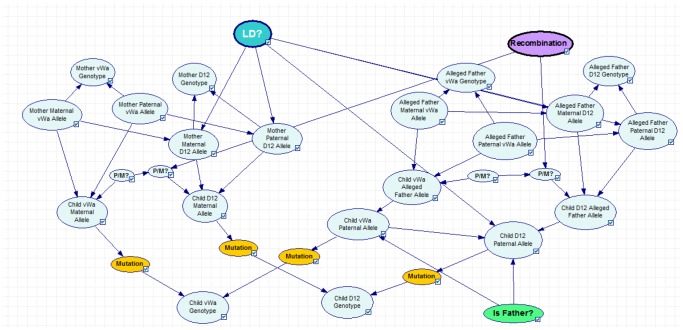
Bayesian network describing a paternity case. The *Recombination* node contains the probability for a recombination to occur, i.e., the recombination rate. The nodes P/M tell whether the vWa paternal or maternal allele is inherited. The LD node is connected to the paternal and maternal allele nodes and decides whether or not to use conditional allele probabilities. Furthermore, the node *Is Father?* contains the different hypotheses.

**Figure 3:**
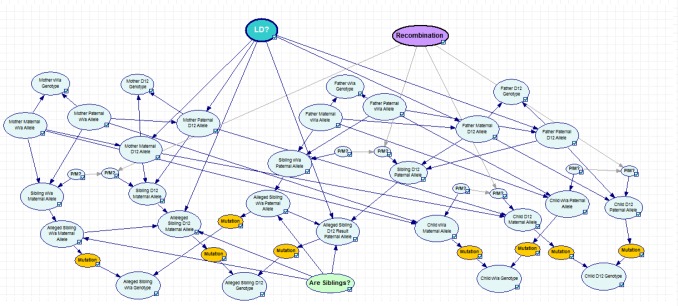
Bayesian network describing a sibling case. The nodes P/M tell whether the vWa paternal or maternal allele is inherited. The P/M nodes connected to the D12S391 allele also contains the recombination frequency. The LD node is connected to the paternal and maternal allele nodes and decides whether or not to use conditional allele probabilities. Furthermore, the node *Are Siblings?* contains the different hypotheses.

Two different scenarios were considered; a regular paternity case, Fig. 2 and a case of disputed siblingship, Fig. 3. For each network the user can vary recombination rate, decide whether to use conditional allele probabilities, based on Table 2, or allele frequencies, see Table 1. In the paternity network the user can decide whether to instantiate the mother's genotypes (trio) or to leave them unknown (duo), i.e. to use the allele frequencies. In the sibling network the hypotheses compare whether the two persons are unrelated or full siblings. (A separate network was also constructed for a halfsibling case when the siblings are known to share the same mother, see Fig. S1.) The parents' genotypes can be instantiated if available, otherwise allele frequencies will be used. The network in Fig. 1 is in principle equal to the one described by Taroni *et al.* for a paternity case [15]. The main differences lie in the existence of a *Recombination* node as well as an *LD* node. The *Recombination* node describes the probability for a cross-over to occur, i.e. the recombination rate. Also for each possible inheritance of a D12S391 allele, the *P/M* nodes transmit whether the **Paternal** or **Maternal** vWa allele have been passed on. The *LD* node is also connected to each possible inheritance of a D12S391 allele. If the *LD* node is instantiated to **Yes**, conditional allele probabilities will be used. The *Mutation* nodes contain a transition matrix. In this study a simple mutation model was used, where each transition has an equal probability of occurring, i.e. μ/(n−1), where μ is the mutation rate and n is the number of alleles. Mutation rates for each locus were obtained from a local database. The *Child Paternal Allele* (CPA) nodes are subject to the *Hypothesis* node (Either *Is Father* or *Are Siblings* depending on the network), with states **Yes** and **No**. The *Hypothesis* node will display the posterior probabilities for the given relationships. The tables for the CPA nodes are based on the Alleged father given that he is the father and the allele frequencies if he is not the father. Also, if the *LD* node is set to **Yes**, conditional allele probabilities for the D12S391 allele will be used. (Please see user manual for a more complete description.)

## Results

The networks were tested on a selection of real cases where the likelihood ratio (LR), assuming marker independence, had already been calculated using the software Familias [10]. In addition an attempt was made to create a worst-case-scenario (WCS) regarding linkage disequilibrium, i.e.,selecting the haplotypes where the observed haplotype frequencies deviated maximally from the expected haplotype frequencies, see Table S1 and S2. The genotypes used in the WCS include rare alleles and as a consequence also often unobserved haplotypes. Table 3 describes the results from the likelihood ratio calculations. Each case was investigated using three different methods. The method denoted M1 in Table 3 is equivalent to the most commonly used approach in forensic laboratories, where the markers vWa and D12S391 are considered to be independent, i.e. recombination rate of 50%, and allele frequencies are utilized. In the two remaining methods, denoted M2 and M3 in Table 3, a recombination rate of 9% was used in accordance with previous studies by Budowle *et al.* [3]. In addition, the decision of whether to use conditional allele probabilities were evaluated, using in M2 allele frequencies (Table 1) and in M3 conditional allele probabilities (Table 2). Quotients between the LR values obtained using each method are included in Table 3. (Note that M2 is not relevant in standard duo/trio cases since recombination alone does not effect the statistical calculations)

**Table 3:** Comparison of calculated likelihood ratios (LR) based on the genotype data from STR loci vWa and D12S391, on a selection of real cases.

Case id	M1	M2	M3	Comparison	LR_M1_/LR_Comparison_	LR_M1_/LR_M2_	LR_M2_/LR_M3_
***Duos***							
1	3.608	3.608	1.909	3.78	0.954	-	1.89
2	3.038	3.038	2.769	3.099	0.98	-	1.097
3	25.455	25.455	35.036	24.243	1.05	-	0.727
4	8.723	8.723	9.638	9.447	0.923	-	0.905
5	8.93	8.93	10.792	9.036	0.988	-	0.827
6	39.487	39.487	51.46	41.563	0.95	-	0.767
7	11.761	11.761	11.859	10.631	1.106	-	0.992
8	2.943	2.943	3.721	2.66	1.106	-	0.791
9	5.956	5.956	6.457	6.463	0.922	-	0.922
10	6.81	6.81	8.912	6.815	0.999	-	0.764
WCS	750.879	750.879	308.597	404	1.859	-	2.433
***Trios***							
11	5.567	5.567	5.055	5.239	1.063	-	1.101
12	96.809	96.809	107.696	89.208	1.085	-	0.899
13	11.626	11.626	7.026	10.834	1.073	-	1.655
14	87.652	87.652	52.191	54.74	1.601	-	1.679
15	8.32	8.32	7.772	9.498	0.876	-	1.071
16	29.479	29.479	21.491	28.919	1.019	-	1.372
17	6.214	6.214	7.347	6.624	0.938	-	0.846
18	11.234	11.234	9.624	11.628	0.966	-	1.167
19	24.483	24.483	33.811	24.8	0.987	-	0.724
20	11.635	11.635	12.358	10.827	1.075	-	0.941
WCS	2917.855	2917.855	736.46	2130	1.37	-	3.962
***Siblings***							
21	9.917	7.097	9.732	9.766	1.015	1.397	0.729
22	0.264	0.287	0.296	0.405	0.652	0.92	0.97
23	38.841	62.98	71.993	38.331	1.013	0.617	0.875
24	0.351	0.339	0.314	0.34	1.032	1.035	1.08
25	1.331	1.584	1.439	1.331	1	0.84	1.101
26	0.46	0.621	0.633	0.455	1.011	0.741	0.981
27	0.378	0.354	0.363	0.38	0.995	1.068	0.975
28	0.83	0.622	0.612	0.815	1.018	1.334	1.016
29	8.61	10.962	11.92	9.1278	0.943	0.785	0.92
30	13.772	19.825	19.367	13.763	1.001	0.695	1.024
WCS	200.938	298.868	134.619	115.694	1.737	0.672	2.22

Three different methods have been used, denoted M1, M2 and M3. M1: 50% recombination rate, LD not considered; M2: 10% recombination, LD not considered; M3: 10% recombination, LD taken into consideration. The column *Comparison* is the LR obtained using the software Familias with the *standard* Norwegian population database. WCS. abbreviates Worst Case Scenario and attempts to simulate a case where the likelihood ratios should differ the most due to linkage disequilibrium. The columns to the right display three relevant quotients for each case; Note that the LR calculated using M2 and the quotient LR_M1_/LR_M2_ is only relevant in the non-paternity cases, since recombination alone will not effect the likelihoods for these cases.

To further test the method, we also created a network where instead of using data from D12S391 and vWa we used data from two other closely located markers, D5S818 and CSF1PO (Table 4). A recombination rate of 0.3 was used, close to the value obtained using any of the mapping functions. The results reveal that, even when comparing two markers accepted to be in LE, discrepancies can be detected. Future studies should be conducted involving markers known to be in LD. Our network can of course be extended to include more linked markers in LD.

**Table 4:** Comparison of calculated likelihood ratios (LR) based on the genotype data from STR loci D5S818 and CSF1PO, on a selection of cases.

Case id	M1	M2	M3	Comparison	LR_M1_/LR_Comparison_	LR_M1_/LR_M2_	LR_M2_/LR_M3_
***Duos***							
1	1.4632	1.4632	1.5058	1.4215	1.029	-	0.972
2	1.062	1.062	1.034	1.037	1.024	-	1.027
3	4.84	4.84	7.998	5.176	0.935	-	0.605
4	395.668	395.668	362.636	485.808	0.814	-	1.091
5	9.598	9.598	9.016	10.246	0.937	-	1.065
6	74.489	74.489	80.653	100.604	0.74	-	0.924
7	8.072	8.072	8.013	7.734	1.044	-	1.007
8	19.193	19.193	20.172	20.491	0.937	-	0.951
9	49.869	49.869	42.537	55.005	0.907	-	1.172
10	77.215	77.215	121.659	114.202	0.676	-	0.635
W.C.S.	1520.143	1520.143	11036.52	3656	0.416	-	0.138
***Trios***							
11	40.007	40.007	64.944	48.709	0.821	-	0.616
12	11.369	11.369	11.272	9.947	1.143	-	1.009
13	5.746	5.746	5.577	8.65	0.664	-	1.03
14	101.284	101.284	85.736	63.917	1.585	-	1.181
15	604.62	604.62	383.645	777.506	0.778	-	1.576
16	23.505	23.505	22.616	25.496	0.922	-	1.039
17	76.821	76.821	52.45	87.727	0.876	-	1.465
18	1838.249	1838.249	1964.408	2138.332	0.86	-	0.936
19	394.116	394.116	216.855	346.241	1.138	-	1.817
20	53.305	53.305	69.457	66.978	0.796	-	0.767
W.C.S.	139.278	139.278	709.883	138.139	1.008	-	0.196
***Siblings***							
21	6.218	5.808	5.02	6.742	0.922	1.071	1.157
22	0.906	0.906	0.93	0.696	1.301	1	0.974
23	4.202	3.99	3.92	3.75	1.121	1.053	1.018
24	3.632	3.343	3.499	2.856	1.272	1.086	0.955
25	0.247	0.265	0.139	0.255	0.968	0.935	1.903
26	6.407	6.407	6.165	4.441	1.443	1	1.039
27	0.158	0.177	0.171	0.154	1.022	0.892	1.037
28	0.256	0.256	0.157	0.16	1.596	1	1.636
29	0.5	0.5	0.548	0.25	2.001	1	0.912
30	0.758	0.758	0.727	0.563	1.347	1	1.043
W.C.S.	23254.65	24999	40649.41	93209.73	0.249	0.93	0.615

Three different methods have been used, denoted M1, M2 and M3. M1: 50% recombination rate and LD not considered. M2: 30% recombination and LD not considered, M3: 30% recombination and LD taken into consideration. The column *Comparison* is the LR obtained using the software Familias with the *standard* Norwegian population database. WCS abbreviates Worst Case Scenario and attempts to simulate a case where the likelihood ratios should differ the most due to linkage disequilibrium. The columns to the right display three relevant quotients for each case; Note that the LR calculated using M2 and the quotient LR_M1_/LR_M2_ is only relevant in the non-paternity cases, since recombination alone will not effect the likelihoods for these cases.

## Discussion

We have demonstrated the application of Object Oriented Bayesian Networks in modeling linkage, linkage disequilibrium and mutations in cases of disputed genetic relatedness. As an example, we present data from a pair of STR markers, vWA and D12S391, recently studied with regards to possible linkage disequilibrium. Two different networks were created to investigate a selection of actual cases as well as fictional, see Worst Case Scenarios in Table 3. The small differences in calculated LRs between method M1 (not considering linkage and LD) and the *Comparison* are due to the use of slightly different allele frequency databases, where the *Comparison* LR has been calculated using a Norwegian population database utilized in routine casework. However, it is notable that the differences between the results using any of M1, M2 (10% recombination rate and LD not considered) or M3 (10% recombination rate and LD is considered) are in many cases comparable to the differences between M1 and the *Comparison* methods. Consequently, the differences between method M2 and M3, allele frequencies versus conditional allele probabilities, can perhaps be considered as merely a small bias in the estimation of allele frequencies.

Since linkage has previously been measured between vWa and D12S391, the most important concern of this paper is to evaluate the effect of using conditional allele probabilities as measured by the quotient between the LR values obtained using methods M3 and M2, see Table 3. None of the real cases display a quotient LR_M2_/LR_M3_ larger than 2, and for most of the cases the quotient is close to 1. Also, the Worst Case Scenarios do not display quotients larger than 4. We should of course always expect some differences since no data will indicate exact linkage equilibrium (Table 4). Whereas our study has only included a small selection of real cases, we are aware that larger studies considering hundreds of cases should be conducted and also that our results, concerning possible LD between vWA and D12S391, are partly anecdotal. A recent paper by Gill *et al.* provides further evidence and discussion on the matter [2].

Haplotype frequencies are generally hard to estimate as genotype data does not normally indicate which chromosome, i.e. paternal or maternal, each allele is located on. New methods, such as mass-sequencing provide means to determine each chromosomal setup, but given current forensic casework, using STR markers, one might instead rely on the massive amount of available data from families (trios mainly) where the haplotypes from the children can, in most cases, be unambiguously determined, as long as the possibility of mutation is disregarded. In our study we used 444 phased unrelated children, i.e. 888 haplotypes, to determine the observed as well as expected haplotype frequencies. We observed 100 of a total of 128 possible haplotypes. An important consideration is if this is enough material for a reliable estimation of population haplotype frequencies? In particular, can we reliably estimate the probability of observing a haplotype that has not been observed in the database? The same dilemma exists when previously unseen or new alleles are observed in regular genotyping, but for haplotypes one may use allele frequencies to construct a reasonable guess at a probability. Our formula contains a parameter λ which loosely corresponds to the pseudo-counts often used in the estimation of population allele frequencies. Although a value for λ might be estimated for data, we have simply used λ = 1. This gives the initial estimates, constructed as products of allele frequencies, the same weight as a single haplotype observation, leading to fairly small estimates of conditional probabilities for unobserved haplotypes.

## Conclusions

An imminent practical concern for forensic laboratories using closely located STR markers, such as the pair studied in this paper, is how computations should be performed with such data. One issue is whether linkage must be taken into account. Though statistical calculations in regular paternity cases is not affected by linkage and disputed paternities make up the majority of cases for most labs, we believe that in sibling cases and other more extended relationships, linkage should be taken into account. We recommend that forensic labs perform sensitivity calculations and/or simulations to investigate the effect of recombination rate, especially in kinship analyses and deficient paternity cases. The recently released software FamLink provides features to perform such analyses [24]. In addition to STR markers, our model can easily be extended to accommodate SNP data. In fact, the networks available at our repository are able to handle diallelic markers, but to process high throughput data a more automated system is needed.

The other major concern, besides recombination, is whether to use conditional allele probabilities, i.e. to account for linkage disequilibrium. All calculations are affected by the use of such probabilities, even standard paternity and match probability calculations. The effect on the marker pair vWA/D12S391 is, according to our results, reasonably small. In addition, the marker pair D5S818/CSF1PO displays equal deviation from expectation, further corroborating results in previous studies. Moreover, our implementation heavily depends on the estimates of conditional allele probabilities, which are currently fairly uncertain. We have illustrated how estimates can be generated based on data from trios, but clearly much larger datasets are needed to reduce the uncertainty. Furthermore, other models to approach the problem with unseen haplotypes should be considered.

Nevertheless, this paper demonstrates how software implementing Object Oriented Bayesian Networks can be used to assemble and code models reasonably quickly, and how these models can subsequently be used to explore complex questions about the interplay between genetic phenomena such as linkage, LD, and mutations. The models can then in fact be used for relevant computations in actual cases. We present Bayesian networks for two basic relationships, available at http://arken.umb.no/~dakl/BayesianNetworks/, which can be used as prototypes for investigations of linkage and linkage disequilibrium for pairs of closely located STR markers.

## Supporting Information

Figure S1 Bayesian network describing a sibling case, where the children are known to share the same mother. The nodes P/M tell whether the vWa paternal or maternal allele is inherited. The P/M node connected to the D12S391 allele also contains the recombination frequency. The LD node is connected to the paternal and maternal allele nodes and decides whether or not to use conditional allele frequencies. Furthermore, the node *Are Siblings?* contains the different hypotheses.
(DOC)

Table S1 Observed haplotype frequencies.
(DOC)

Table S2 Expected haplotype frequencies.
(DOC)
