# Supplementary material for: Using Object Oriented Bayesian Networks to Model Linkage, Linkage Disequilibrium and Mutations between STR Markers
Source: PLoS One. 2012 Sep 11;7(9):e43873. doi: 10.1371/journal.pone.0043873 (PMC3439468; doi:10.1371/journal.pone.0043873)
Supplement: Figure S1 — Bayesian network describing a sibling case, where the children are known to share the same mother. The nodes P/M tell whether the vWa paternal or maternal allele is inherited. The P/M node connected to the D12S391 allele also contains the recombination frequency. The LD node is connected to the paternal and maternal allele nodes and decides whether or not to use conditional allele frequencies. Furthermore, the node Are Siblings? contains the different hypotheses. (DOC) [file pone.0043873.s001.doc]

# Supplementary information to *Using Bayesian Networks to model linkage and linkage disequilibrium between STR markers by Kling et al.*

***Figure S1.*** Bayesian network describing a sibling case, where the children are known to share the same mother. The nodes P/M tell whether the vWa paternal or maternal allele is inherited. The P/M node connected to the D12S391 allele also contains the recombination frequency. The LD node is connected to the paternal and maternal allele nodes and decides whether or not to use conditional allele frequencies. Furthermore, the node *Are Siblings?* contains the different hypotheses.
