# Supplementary material for: Using Object Oriented Bayesian Networks to Model Linkage, Linkage Disequilibrium and Mutations between STR Markers
Source: PLoS One. 2012 Sep 11;7(9):e43873. doi: 10.1371/journal.pone.0043873 (PMC3439468; doi:10.1371/journal.pone.0043873)
Supplement: Table S1 — Observed haplotype frequencies. (DOC) [file pone.0043873.s002.doc]

# Supplementary information to *Using Bayesian Networks to model linkage and linkage disequilibrium between STR markers by Kling et al.*

**Table S1. Observed haplotype probabilities.**

|  | **14** | **15** | **16** | **17** | **18** | **19** | **20** | **21** |
| --- | --- | --- | --- | --- | --- | --- | --- | --- |
| **15** | 0.003379 | 0.002253 | 0.007885 | 0.013514 | 0.011258 | 0.005629 | 5.01E-07 | 5.56E-08 |
| **16** | 2.25E-06 | 0.002252 | 0.004505 | 0.009007 | 0.005629 | 0.001127 | 2.57E-07 | 2.85E-08 |
| **17** | 0.01576 | 0.00451 | 0.033776 | 0.034914 | 0.018025 | 0.013513 | 3.38E-03 | 1.57E-07 |
| **17.3** | 1.35E-06 | 1.11E-06 | 0.003378 | 0.005629 | 0.003378 | 0.001126 | 1.54E-07 | 1.71E-08 |
| **18** | 0.015767 | 0.014639 | 0.038292 | 0.064185 | 0.043913 | 0.01577 | 1.13E-03 | 2.45E-07 |
| **18.3** | 0.001126 | 0.001126 | 0.001128 | 0.004504 | 0.005627 | 1.56E-06 | 1.54E-07 | 1.71E-08 |
| **19** | 0.012384 | 0.004508 | 0.024773 | 0.029284 | 0.022521 | 0.013511 | 1.22E-06 | 1.36E-07 |
| **19.3** | 1.13E-06 | 0.001126 | 0.003377 | 0.005628 | 0.001127 | 1.3E-06 | 1.28E-07 | 1.43E-08 |
| **20** | 0.00676 | 0.012382 | 0.018024 | 0.028159 | 0.027021 | 0.014636 | 1.13E-03 | 1.37E-07 |
| **21** | 0.011259 | 0.011257 | 0.023647 | 0.030407 | 0.014646 | 0.010136 | 1.13E-03 | 1.30E-07 |
| **22** | 0.006761 | 0.009008 | 0.022525 | 0.04391 | 0.019148 | 0.011262 | 2.25E-03 | 1.46E-07 |
| **23** | 0.011258 | 0.004507 | 0.021395 | 0.025904 | 0.015769 | 0.011259 | 1.13E-03 | 1.12E-03 |
| **24** | 0.003378 | 0.004503 | 0.010133 | 0.010137 | 0.007883 | 0.002254 | 4.37E-07 | 4.85E-08 |
| **25** | 0.001126 | 0.001126 | 0.001127 | 0.003378 | 0.001127 | 0.001126 | 1.03E-07 | 1.14E-08 |
| **26** | 3.38E-07 | 2.78E-07 | 0.002251 | 1.18E-06 | 0.001126 | 3.89E-07 | 3.85E-08 | 4.28E-09 |
| **27** | 2.25E-07 | 1.85E-07 | 5.48E-07 | 0.001126 | 5.02E-07 | 0.001125 | 2.57E-08 | 2.85E-09 |

Alleles from vWa is represented in each column while alleles from D12S391 is represented in each row. To account for unseen haplotypes, probabilities were estimated using a flat Dirichlet distribution, based on the number of observation for haplotype.
