# Supplementary material for: Using Object Oriented Bayesian Networks to Model Linkage, Linkage Disequilibrium and Mutations between STR Markers
Source: PLoS One. 2012 Sep 11;7(9):e43873. doi: 10.1371/journal.pone.0043873 (PMC3439468; doi:10.1371/journal.pone.0043873)
Supplement: Table S2 — Expected haplotype frequencies. (DOC) [file pone.0043873.s003.doc]

# Supplementary information to *Using Bayesian Networks to model linkage and linkage disequilibrium between STR markers by Kling et al.*

**Table S2. Expected haplotype frequencies.**

|  | **14** | **15** | **16** | **17** | **18** | **19** | **20** | **21** |
| --- | --- | --- | --- | --- | --- | --- | --- | --- |
| **15** | 0.003907 | 0.003215 | 0.009496 | 0.013601 | 0.008705 | 0.004501 | 4.45E-04 | 4.95E-05 |
| **16** | 0.002004 | 0.001649 | 0.00487 | 0.006975 | 0.004464 | 0.002308 | 2.28E-04 | 2.54E-05 |
| **17** | 0.01102 | 0.009067 | 0.026784 | 0.038362 | 0.024552 | 0.012694 | 1.26E-03 | 1.39E-04 |
| **17.3** | 0.001202 | 0.000989 | 0.002922 | 0.004185 | 0.002678 | 0.001385 | 1.37E-04 | 1.52E-05 |
| **18** | 0.017232 | 0.014178 | 0.04188 | 0.059984 | 0.03839 | 0.019849 | 1.96E-03 | 2.18E-04 |
| **18.3** | 0.001202 | 0.000989 | 0.002922 | 0.004185 | 0.002678 | 0.001385 | 1.37E-04 | 1.52E-05 |
| **19** | 0.009518 | 0.007831 | 0.023131 | 0.033131 | 0.021204 | 0.010963 | 1.08E-03 | 1.20E-04 |
| **19.3** | 0.001002 | 0.000824 | 0.002435 | 0.003487 | 0.002232 | 0.001154 | 1.14E-04 | 1.27E-05 |
| **20** | 0.009618 | 0.007913 | 0.023375 | 0.033479 | 0.021427 | 0.011079 | 1.10E-03 | 1.22E-04 |
| **21** | 0.009117 | 0.007501 | 0.022157 | 0.031736 | 0.020311 | 0.010502 | 1.04E-03 | 1.15E-04 |
| **22** | 0.010219 | 0.008408 | 0.024836 | 0.035572 | 0.022766 | 0.011771 | 1.16E-03 | 1.29E-04 |
| **23** | 0.008215 | 0.006759 | 0.019966 | 0.028597 | 0.018302 | 0.009463 | 9.36E-04 | 1.04E-04 |
| **24** | 0.003406 | 0.002803 | 0.008279 | 0.011857 | 0.007589 | 0.003924 | 3.88E-04 | 4.31E-05 |
| **25** | 0.000801 | 0.000659 | 0.001948 | 0.00279 | 0.001786 | 0.000923 | 9.13E-05 | 1.01E-05 |
| **26** | 0.000301 | 0.000247 | 0.00073 | 0.001046 | 0.00067 | 0.000346 | 3.42E-05 | 3.80E-06 |
| **27** | 0.0002 | 0.000165 | 0.000487 | 0.000697 | 0.000446 | 0.000231 | 2.28E-05 | 2.54E-06 |

Alleles from vWa is represented in each column while alleles from D12S391 is represented by each row. The expected haplotype frequencies are calculated by multiplying the frequencies of the alleles in each haplotype configuration.
